# Phosphoglucomutase Is Not the Target for Galactose Toxicity in Plants

**DOI:** 10.3389/fpls.2020.00167

**Published:** 2020-02-28

**Authors:** Martina Althammer, Constantin Blöchl, Roland Reischl, Christian G. Huber, Raimund Tenhaken

**Affiliations:** ^1^ Molecular Plant Physiology, Department of Biosciences, University of Salzburg, Salzburg, Austria; ^2^ Bioanalytical Research Labs, Department of Biosciences, University of Salzburg, Salzburg, Austria

**Keywords:** sugar toxicity, UDP-sugar pyrophosphorylase, salvage pathway nucleotide sugars, cytosolic phosphoglucomutase, galactose-1-phosphate

## Abstract

Plants synthesize a number of different oligomeric or polymeric sugars containing galactose. During growth and development some of these carbohydrates are metabolized or remodeled releasing galactose as a breakdown product. All plants have established recycling pathways for such sugars, for which they seem to have a limited capacity to cope with. Exceeding these limits results in sugar toxicity, which is observed already at concentrations as low as 1 mmol·l^−1^ for galactose. The mechanism of galactose toxicity is poorly understood but it seems plausible that the enzymes involved in carbohydrate metabolism also might be the targets responsible for the adverse effects. Data from yeast and bacteria suggests that the enzyme phosphoglucomutase (PGM) is inhibited by galactose-1-phosphate. To test this hypothesis for plants we expressed recombinant cytosolic PGM3 from Arabidopsis in *E. coli*. Intriguingly, the enzyme was not inhibited by galactose-1-phosphate at physiological concentrations. Furthermore, PGM3 did not convert galactose-1-phosphate to galactose-6-phosphate, which was suggested as the inhibitory mode of action in yeast. In addition, metabolite levels in Arabidopsis roots were analyzed for their galactose-1-phosphate concentration by means of GC–MS. Seedlings grown on MS-media with sucrose contained less than 10 nmol·g FW^−1^ of galactose-1-phosphate. However, seedlings from plates, in which the sucrose was replaced by galactose, showed a strong increase of Gal-1-P to levels of up to 200 nmol·g FW^−1^.

## Introduction

Galactose is an abundant sugar in plants, fungi and animals. The *de novo* synthesis involves the formation of UDP-glucose, which is epimerized to UDP-galactose by the activity of UDP-glucose-4-epimerase (Seifert et al., 2002). Beside the *de novo* pathway, which always leads to nucleotide sugars, most organisms have developed a recycling pathway for galactose. Lactose uptake in humans leads to high amounts of galactose as result of lactose cleavage. Individuals with a mutation in the galactose utilization pathway develop the disease galactosemia already in newborns (Timson, 2016). In plants, galactose is released from cell wall turnover or arabinogalactan protein break-down (Takeuchi and Komamine, 1980; Tenhaken, 2015). Furthermore, plants accumulate oligosaccharides of the raffinose-family oligosaccharides (RFO) under stress or they accumulate high amounts as seed storage carbohydrates (Elsayed et al., 2014; Gangl and Tenhaken, 2016). RFOs are produced from sucrose, to which one or more galactose residues are attached. During seed germination, RFOs are broken down to galactose and sucrose. Galactose must enter a recycling pathway to be converted to UDP-galactose (Gangl and Tenhaken, 2016). Given the many situations, in which organisms come in contact with galactose, it is surprising that the addition of low concentrations of galactose already exerts toxic effects on these organisms.

In humans and fungi, including yeast, galactose utilization consists of three steps often called the Leloir pathway (Paladini and Leloir, 1952). First, galactose is phosphorylated by galactokinase to yield galactose-1-phosphate (Gal-1P). This molecule subsequently reacts with UDP-glucose and the uridyl-residue is transferred to Gal-1P. As a consequence, UDP-galactose and glucose-1-phosphate (Glc-1P) are the products of this reaction. A UDP-glucose-4-epimerase can later adjust the equilibrium between UDP-galactose and UDP-glucose. Glc-1P may enter glycolysis after conversion to glucose-6-phosphate, (Glc-6P) catalyzed by the enzyme phosphoglucomutase (PGM). The Leloir pathway has been found to occur in mammals and fungi (Segawa and Fukasawa, 1979; Timson, 2016; Schuler et al., 2018). Proof of the Leloir pathway in plants is still widely lacking. (Dai et al., 2006) found evidence for this pathway in soybean and melons. The key enzyme of the Leloir pathway, the galactose 1-phosphate uridyltransferase (GALT) was previously purified and characterized from the red algae *Galdieria sulphuraria* (Gross and Schnarrenberger, 1995) and *Gracilaria changii* (Siow et al., 2012) suggesting a functional Leloir pathway in this plant lineage. Higher plants however have developed a new enzyme, called UDP-sugar pyrophosphorylase, which can convert many different sugar-1-phosphates including Gal-1P into the respective UDP-sugars (Kotake et al., 2004). This enzyme has not yet been identified in humans and yeast.

The molecular basis for galactose toxicity in any organism is still under debate. Genetic data suggest that a nonfunctional galactokinase strongly reduces the toxic effect of galactose in humans. A knockout in galactokinase reduces the toxic effect of Gal and has led to the development of small molecule galactokinase inhibitors as potential drugs to treat galactosemia (Lai et al., 2014). The formation of Gal-1P is also a pre-requisite for toxicity in plants and fungi (Egert et al., 2012; Schuler et al., 2018). A galactokinase knockout plant has been recently identified (Egert et al., 2012). It has lost the toxicity phenotype observed in WT plants. Yeast mutants, in which genes of the Gal utilization were either disrupted or overexpressed, showed growth inhibition on Gal-containing media, which can be explained at least partially by Gal-1P accumulation and the inhibition of the enzyme PGM (De Jongh et al., 2008). However, Gal-1P was not directly determined. Instead of Gal-1P galactose uptake and growth rate of yeast cultures were measured.

It is often assumed that Gal-1P inhibits the enzyme PGM that catalyzes the equilibrium between Glc-1P and Glc-6P (Gitzelmann, 1995; Joersbo et al., 2003; De Jongh et al., 2008). In addition, the bacterial β-PGM from *Lactococcus lactis* co-crystallizes with its inhibitor Gal-1P. From the structural data as well as from biochemical experiments, a possible mode of Gal-1P inhibition of PGM was deduced (Zhang et al., 2005).

A functional genomic study with cytoplasmatic PGMs in Arabidopsis revealed the essential function of PGM for plant growth. Whereas single mutants in one of the two cytoplasmatic isoforms PGM2 or PGM3 are indistinguishable from WT plants, a double k.o. in both isoforms was not viable (Egli et al., 2010). Gene silencing of both cytoplasmatic isoforms of PGM by miRNA showed a strong correlation between PGM activity and normal growth. Strongly silenced plants had severe growth defects and died early (Malinova et al., 2014).

Here, we investigate the accumulation of Gal-1P in plants grown on galactose containing media and dissect the question whether galactose toxicity is mediated by the inhibition of PGM in plants.

## Methods

### Chemicals and Reagents

Glc-1P, Glc-6P, Gal-6P was from Sigma (Vienna, Austria), Gal-1P were purchased from GoldBio (St Louis, USA), Glc-1,6-bP was from Carbosynth (Berkshire, UK) and D-Glucose (U-^13^C6, 99%) was from Cambridge Isotope Laboratories. Pyridine and MSTFA were supplied by Sigma. NADP^+^ and buffers were obtained from Carl-Roth (Karlsruhe, Germany). Glc-6P-DH from *Leuconostoc mesenteroides* was supplied by Megazyme (Wicklow, Ireland) as an ammonium sulfate suspension. Before use, a suitable amount of the enzyme suspension was diluted 1:10 in enzyme buffer and kept on ice until use.

### Plant Material and Growth Conditions

For sugar measurements, seedlings of *Arabidopsis thaliana* (ecotype Columbia; N60000 NASC Nottingham, UK) were used as a wildtype plant. A T-DNA insertion mutant (GabiKat_489_D10) in the galactokinase gene (At3g06580; *galK*) was obtained from NASC and verified by PCR. This mutant was previously characterized by Egert et al. (2012) and shown to have no galactokinase activity. Seeds were surface sterilized by ethanol and incubated on 0.5× MS plates (Basal Salt Mixture, Duchefa #M0245, Haarlem, Netherlands), pH 5.7 (KOH) containing 0.8% plant agar (Duchefa) and 5 mM sucrose. After 7 days they were transferred to 0.5× MS plates, pH 5.7 (KOH) with 0.8% plant agar and 5 mM galactose. Plants were grown in growth chamber under short day conditions at 23°C with 8 h light (approximately 150 μE·m^−2^·s^−1^) and 18°C in the dark. The roots of the seedlings were harvested after 14 days growth on galactose containing medium, frozen in liquid nitrogen and stored at −80°C.

### Sample Extraction and Derivatization

Metabolite extraction was performed using a modification of the method described in Lunn et al. (2006) and Arrivault et al., (2009) with the addition of 400 μl water instead of 200 μl. For determination of the sugar-phosphate level in plants, the dried pellet was dissolved in 300 μl water and split in two equal portions. ^13^C-D-Glc was added as an internal standard to each portion. One of the portions was spiked with 35 µmol·l^−1^ of Gal-1P. After the addition of the internal standard and Gal-1P, the samples were evaporated to dryness using a centrifugal vacuum dryer. For sample preparation, 25 µl of pyridine were added to the dried samples and incubated at 60°C for 30 min. Subsequently, 25 µl of N-Methyl-N-(trimethylsilyl)trifluoroacetamide (MSTFA, Sigma Aldrich) was added to each sample and further incubated at 90°C for 30 min. After 1:2 dilution using n-hexane the samples were analyzed by GC–MS.

### Gas Chromatography

Chromatographic separation was carried out using a FOCUS™ GC instrument, equipped with an AI 3000 autosampler (both Thermo Fisher Scientific, Dreieich, Germany) and a TRACE™ TR-5 capillary column (5% phenyl methyl polysiloxane, 15 m length, 0.25 mm i.d. and 0.25 µm film thickness). The system was operated at a constant carrier gas (helium) flow rate of 1.5 ml·min^−1^. Focusing of the sample was obtained at 120°C for 1 min, followed by a shallow gradient of 1.5°C·min^−1^ from 120 to 195°C and a steep gradient of 20°C·min^−1^from 195 to 300°C, with a final hold of 2 min. Injection of 1 µl sample was performed in split less mode and an inlet temperature of 250°C. The MS transfer line temperature was set to 200°C.

### Mass Spectrometry

The GC instrumentation was hyphenated to a quadrupole MS (DSQ™ II, Thermo Fisher Scientific, Bremen, Germany). Ionization was carried out using electron ionization (EI) in positive mode with an ion-source temperature of 200°C and a detector gain of 3·10^5^. The system was operated in SIM mode recording masses 204.02, 205.99, 257.00, 298.93, 314.97, 387.04 and 428.95 with a dwell time of 10 ms and an *m/z*-width of 1.0. The system was operated using the Xcalibur™ software (2.2 SP1.48, Thermo Fisher Scientific, Waltham, MA, USA). All data were evaluated using Chromeleon Software (version 7.2.8, Thermo Fisher Scientific, Waltham, MA, USA).

### Cloning and Expression of PGM3

The PGM3 gene was amplified by PCR using a cDNA from Arabidopsis WT plant. The amplification was performed with Q5-DNA polymerase (NEB, Frankfurt a.Main, Germany) using the recommended conditions of the manufacturer. Forward primer: CACCATCACCATCACGGAATGGTTTTCAAGGTTTCTACCGTATCC; reverse primer: GTCCAAGCTCAGCTAATTAAGCTTTATGTTATGACGGTGGGG. (98°C 30 s; 4× (98°C 5 s; 56°C 20 s; 72°C 70 s); 20× (98°C 5 s; 66°C 20 s; 72°C 70 s)). A 1.8 kbp fragment was obtained and purified using the PCR-product purification kit (Thermo Fisher Scientific, Vienna). The *E. coli* expression vector pQE30 was cut with Bam*H*I; Hin*D*III (Thermo Fisher Scientific, Vienna) and later inactivated by heat treatment. A Hot Fusion reaction (Fu et al., 2014) was assembled with 30 ng of linearized vector and 50 ng of purified PCR-product in 10 µl volume. Two microliters of the reaction were transformed in chemically competent *E. coli* XL-1 bacteria. A colony containing the PGM3 in the pQE30 vector was selected and the sequence confirmed by Sanger sequencing (Eurofins, Ebersberg, Germany). A preculture was grown at 37°C with vigorous shaking and used to inoculate cultures for recombinant enzyme production. We tested three temperatures (20°C; 30°C; 37°C) and two IPTG concentrations (100 µM; 500 µM) for optimal conditions and finally decided to use 100 µM IPTG and overnight incubation at 20°C. Recombinant protein was purified on a Machery-Nagel (Düren, Germany) Protino 1000 column. The eluted recombinant protein was brought into storage buffer (50 mM Hepes; 50 mM KCl; 2 mM MgCl_2_; 0,5 mM EDTA; 20% Glycerol; pH 7.5 (KOH)) by size exclusion chromatography on a PD10 column (GE Healthcare, Germany). The enzyme was frozen in aliquots in liquid nitrogen and stored at −80°C or used freshly. Freezing does not harm the enzyme activity even after several weeks of storage.

### PGM Enzyme Assay

PGM enzyme activity was measured as the formation of Glc-6P from Glc-1P. Glc-6P was oxidized by glucose-6P-dehydrogenase leading to the formation of NADPH with an increase at 340 nm. A typical enzyme assay consisted of 50 mM triethylammonium bicarbonate (TEAB) (pH 7.0); 2 mM MgCl_2_; 0–2 mM Glc-1P; 10 µM Glc-1,6bP; 0.5 mM NADP^+^; 1 U Glc-6P-DH. The reaction was started by the addition of 0.59 µg recombinant PGM3 to a final volume of 1 ml. To allow the determination of sugar-phosphates from enzyme assays, the reaction was terminated by the addition of 200 µl CHCl_3_/CH_3_OH (3:7, v/v). The mixture was vigorously shacked and incubated for 10 min at −20°C. After centrifugation at 13,000 rpm for 5 min at 4°C, the upper phase was transferred to a new tube and evaporated to dryness using a centrifugal vacuum dryer. The pellet was derivatized and analyzed by GC–MS as described above.

### Data Analysis

Where applicable, data were compared by one-way ANOVA or unpaired t-test using the GraphPad Prism software (version 8.0). Significance levels are marked with ns for non significant or *** for p < 0.001.

## Results

**Figure 1:**
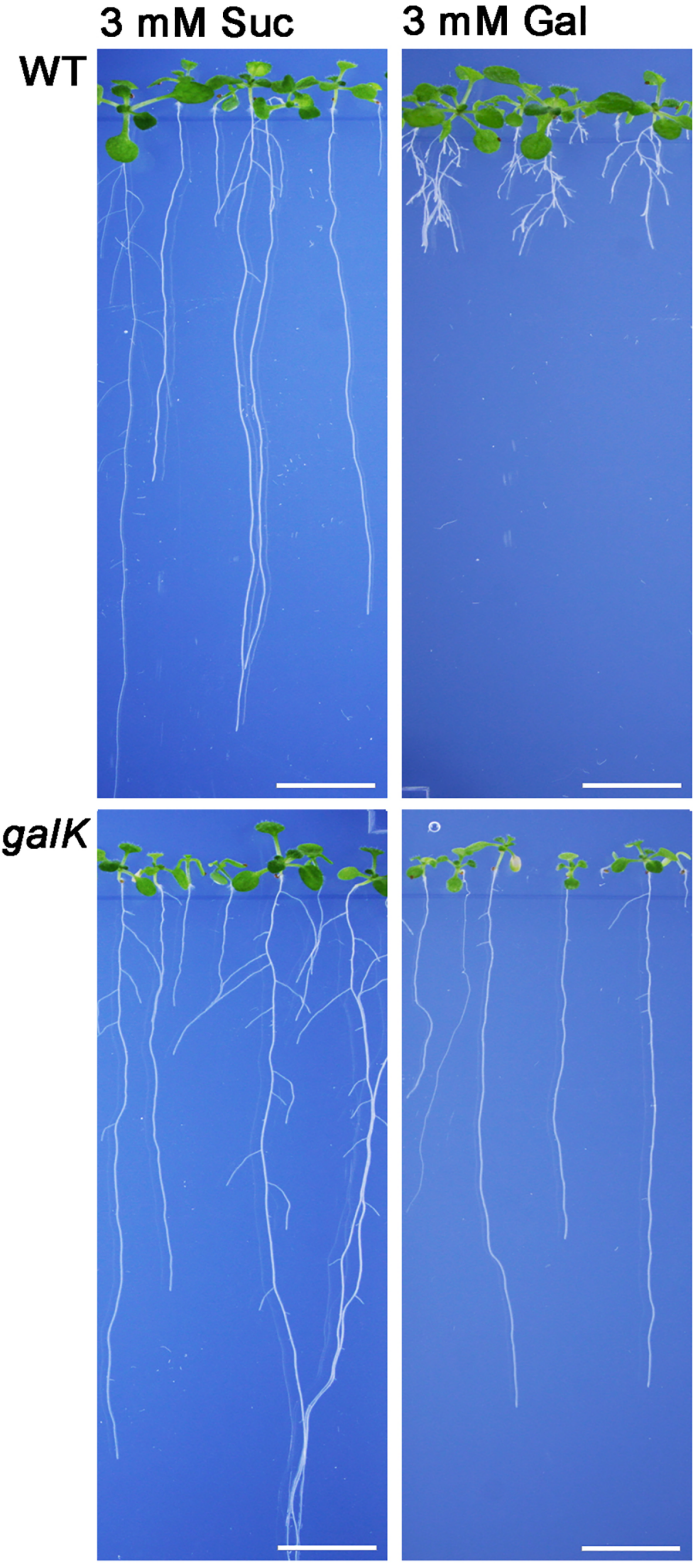
Phenotype of Arabidopsis seedlings on 0.5× MS plates grown for 2 weeks with either 3 mM sucrose (left) or 3 mM galactose (right). WT plants show a severe change in root morphology on plates with galactose. The WT phenotype could be rescued by knocking out the galactokinase gene (*galK)* indicating that galactokinase activity is required for Gal toxicity phenotype. Scale bars: 1 cm.

**Figure 2:**
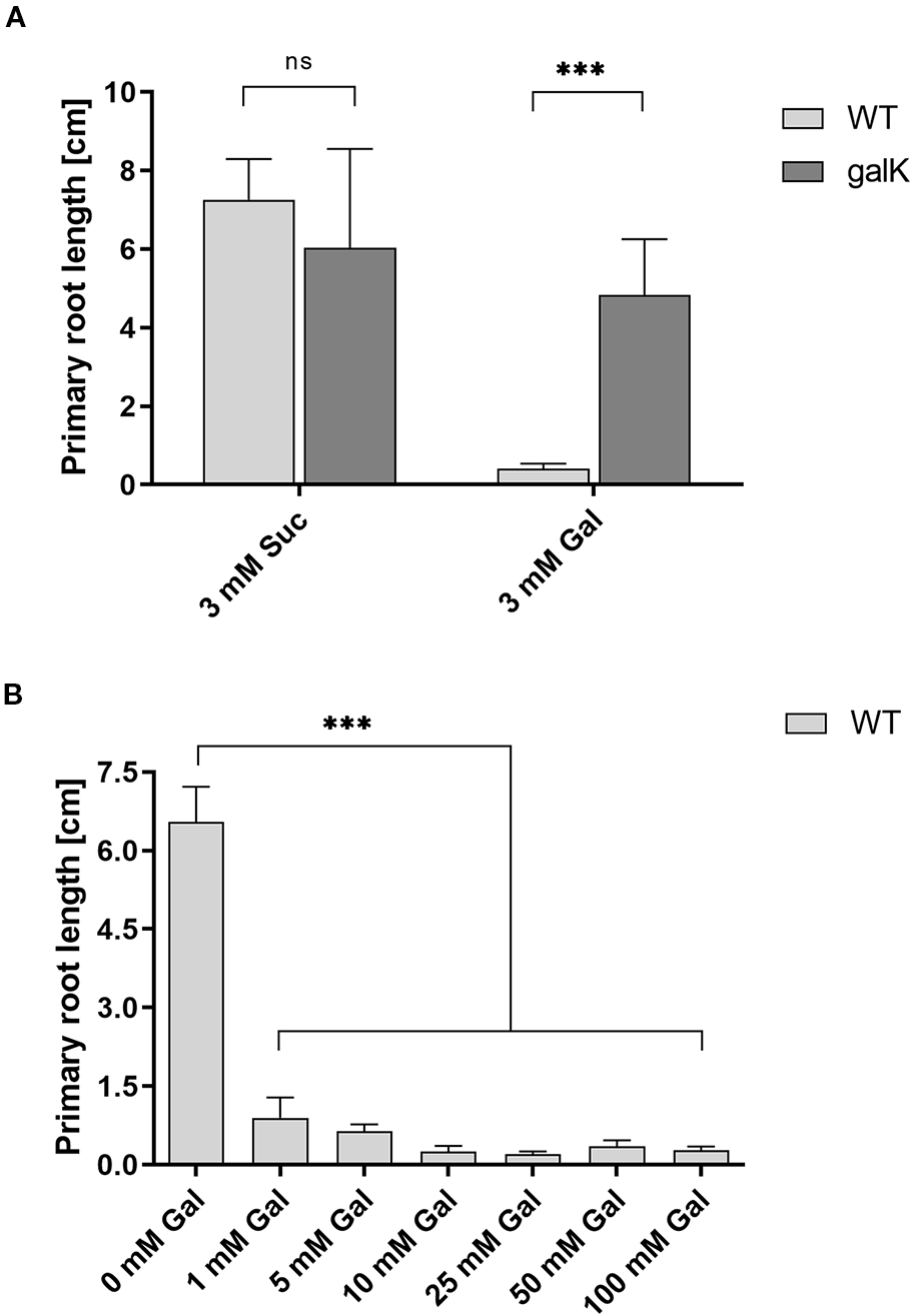
Root length reduction of WT seedlings on agar plates with galactose. **(A)** Primary root length of two weeks old WT seedlings compared to *galK* seedlings on 0.5× MS plates with either 3 mM sucrose or 3 mM galactose. **(B)** Root length of 11 d old WT seedlings growing on increasing galactose concentrations. Values are averages (± SD) of the measurement of six plants using the ImageJ software. Data analysis was performed using one-way Anova and Tukeys multiple comparison test **(A)** or Dunnett’s multiple comparisons test **(B)**; ns, non significant; ***, p <0.001.

**Figure 3:**
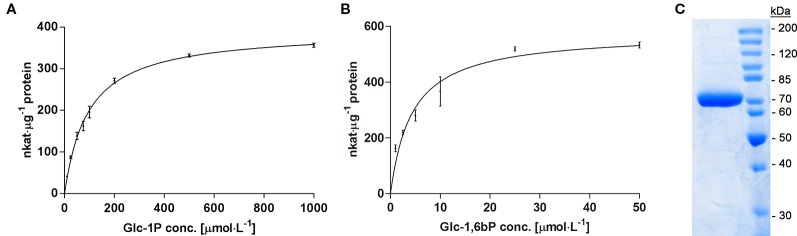
**(A)** Enzyme kinetic of recombinant PGM3 with increasing concentration of the substrate Glc-1P (10 to 1,000 µmol·l^−1^), showing a hyperbolic curve with a K_m_ of 90 µmol·l^−1^. **(B)** Kinetic of PGM3 for the co-substrate Glc-1,6bP that is needed as a catalytic intermediate. The low K_m_ of 4.3 µmol·l^−1^ demonstrates the high affinity of PGM for this co-substrate. PGM Assays were performed at 25°C for 3 min. Values are averages of three independently performed assays (± SD). **(C)** Coomassie-stained SDS-PAGE gel of purified PGM3 showing the high purity of the enzyme preparation. Numbers indicate the molecular weight of proteins standards (Unstained Protein Ladder Thermo Fisher #26614) on a 10% acrylamide gel.

Galactose is an abundant sugar in plant cell wall polymers and cytoplasmic metabolites. Nevertheless, growth of seedlings on MS-plates, containing low concentrations of galactose, shows a strong effect on the root development phenotype (**Figure 1**). The development of the phenotype was concentration dependent. Already an addition of 1 mM Gal causes a strong decrease in root length, shown in **Figure 2B**. The roots were much shorter on galactose plates compared to plates with sucrose (**Figure 2A**). In addition, the branching pattern of the roots differed between media with Gal or sucrose. Mutant plants with an insertional T-DNA (GabiKat_489_D10) in the galactokinase gene were unable to recycle galactose (Egert et al., 2012). They grew very similar to WT plants on MS-plates containing sucrose, demonstrating the necessity of intermediate Gal-1P formation for developing galactose toxicity (**Figure 1**). As galactose is toxic to many organisms including yeast and humans, a number of explanations were hypothesized in the past (Gitzelmann, 1995). Among those was the assumption that the enzyme phosphoglucomutase (PGM) is inhibited by Gal-1P or at least contributes to galactose toxicity by, e.g. the conversion of Gal-1P to Gal-6P. Direct experimental data on this problem was not available for yeast or plants. We therefore cloned the gene for the cytosolic isoform PGM3 from Arabidopsis into a pQE30 expression vector and expressed the recombinant protein with His_6_-tag in *E. coli*. The PGM3 protein (64.5 kDa) was produced in high concentrations and could thus be obtained in high purity after Ni-metal chelate chromatography (**Figure 3C**).

**Figure 4:**
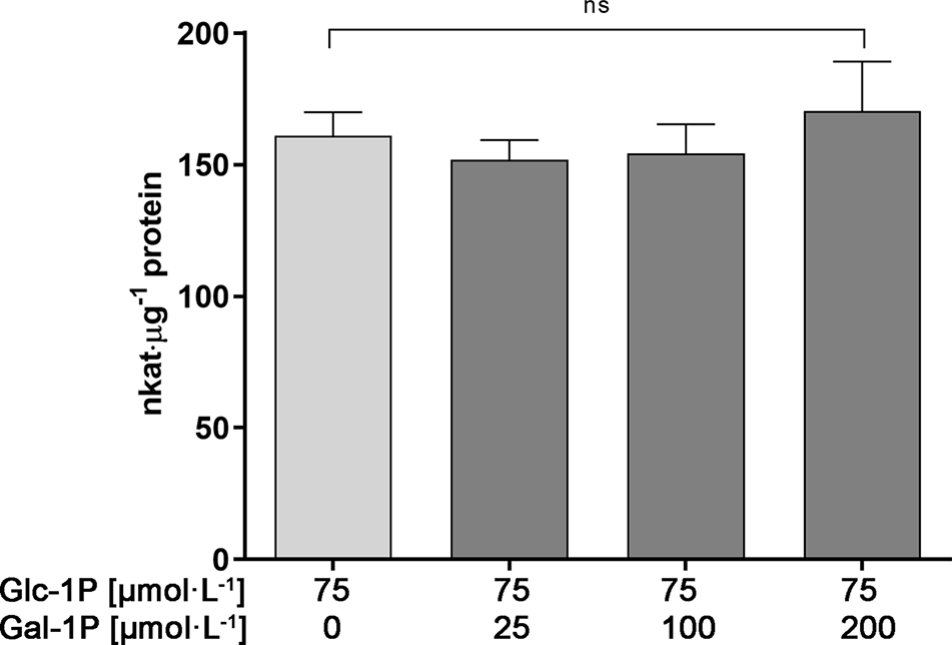
Enzyme activity of PGM3 in the presence of various concentrations of Gal-1P. Even a three-fold surplus of Gal-1P over Glc-1P does not inhibit the PGM3 enzyme activity. Gal-1P concentrations were chosen on the basis of metabolite measurements for Gal-1P found in plants under Gal-feeding conditions. Glc-1P concentration were chosen based on published metabolite data (Arrivault et al., 2009; Szecowka et al., 2013). Mean values of six independently performed measurements are depicted (± SD). Data analysis was performed using one-way Anova and Dunnett’s multiple comparisons test; ns, non significant.

We first tested the possibility that PGM3 is directly inhibited by Gal-1P. An enzyme kinetic with increasing substrate concentrations of Glc-1P is shown in **Figure 3A**. The enzyme exhibited a typical hyperbolic Michaelis-Menten kinetic with a K_m_ value of 90 µmol·l^−1^ and a V_max_ value of 382 nkat·μg^−1^ protein for Glc-1P. Furthermore, PGM needs Glc-1,6-bisphosphate (Glc-1,6bP) as a catalytic intermediate. The kinetic for Glc-1,6bP followed a hyperbolic curve with a very low K_m_-value for Glc-1,6bP of 4.38 µmol·l^−1^ and a V_max_ value of 576 nkat·μg^−1^ protein (**Figure 3B**). Lineweaver-Burk plots showed similar K_m_ values for the substrate Glc-1P as well as for the cosubstrate Glc-1,6bP compared to direct regression analysis of the hyperbolic curves (**Supplemental Data Figure S1**).We added Gal-1P in increasing concentrations (0; 25; 100; 200 µmol·l^−1^) to a PGM standard enzyme assay. None of the chosen concentrations, presumably reflecting physiological concentrations (Arrivault et al., 2009; Szecowka et al., 2013), did inhibit PGM activity (**Figure 4**).

**Figure 5:**
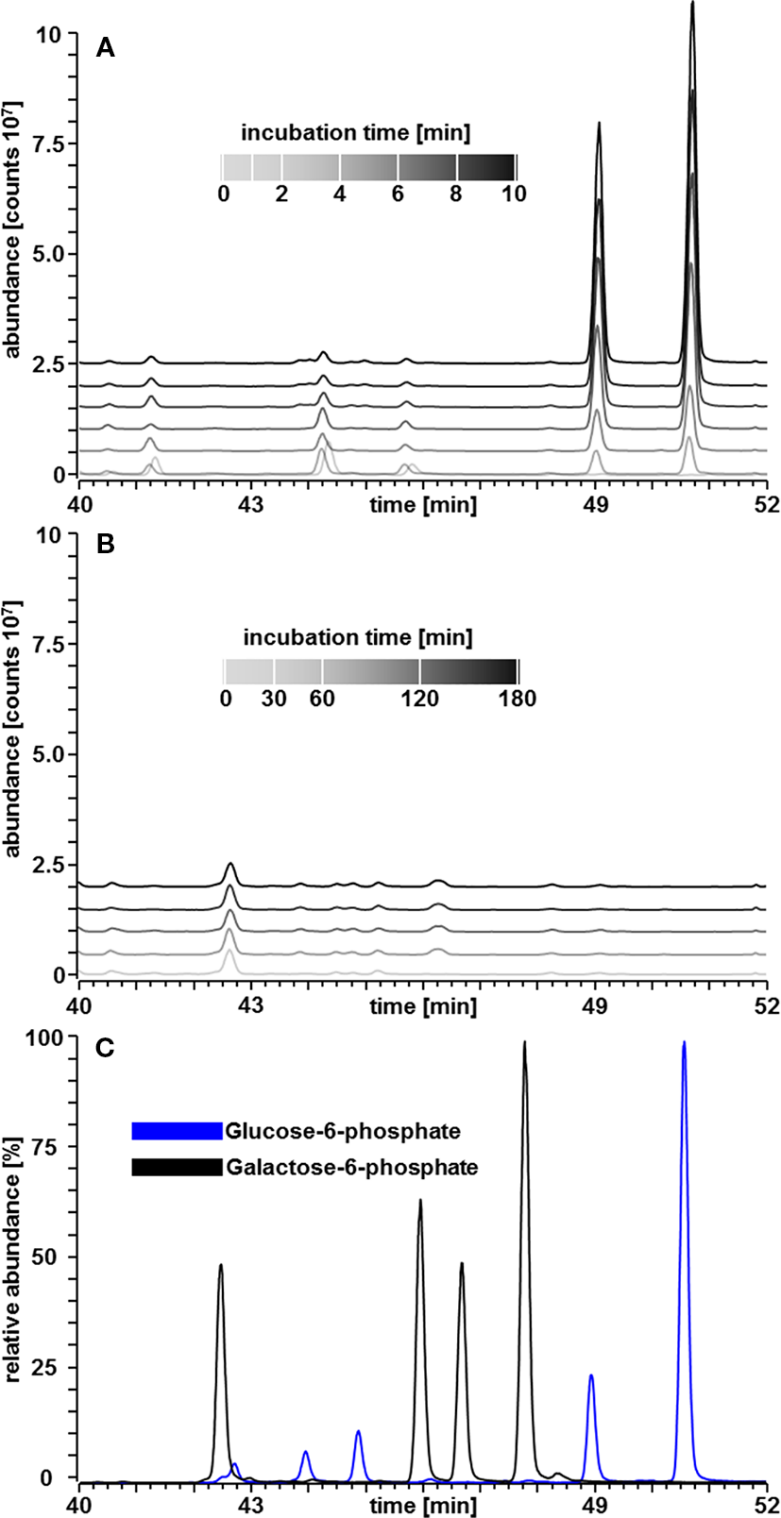
GC–MS chromatograms of sugar phosphates. **(A)** Linear increase in Glc-6P area over time in a PGM assay with Glc-1P (200 µM) as substrate. **(B)** Amounts of Gal-6P in a PGM assay with Gal-1P (200 µM) as substrate instead of Glc-1P. Gal-6P was below the limit of detection of about 35 fmol. **(C)** All four tautomeric forms of Glc-6P and Gal-6P standards, respectively, were readily separated (Martinezcastro et al., 1989).

A second possibility for Gal-1P mediated inhibition of PGM is the conversion of Gal-1P to Gal-6P. In this case, Gal-6P might accumulate and the intermediate formation of a Gal-1,6bP could be inhibitory. In order to test this hypothesis, we established a GC-MS based detection method for sugar-phosphates, which allowed the separation of Glc-1P; Glc-6P; Gal-1P and Gal-6P. A standard curve for Glc-6P and Gal-6P is shown in **Figure 5C**. The method resulted in a linear increase of chromatographic peak areas within the applied concentration range thus confirming that the GC-MS method allowed quantitative determination of these metabolites. We next performed PGM enzyme assays in a volatile buffer and inactivated the enzyme assay by the addition of 200 µl CHCl_3_/CH_3_OH (3:7, v/v) at appropriate time points. The PGM enzyme readily converted Glc-1P into Glc-6P and showed a linear increase in product formation over time (**Figure 5A**; **Table 1**). When Gal-1P was added as a putative substrate, no formation of Gal-6P was observed, even after a prolonged incubation time of 3 h (**Figure 5B**; **Table 1**).

**Table 1:** Product formation of Glc-6P from Glc-1P in an enzyme assay over 10 min measured by GC-MS.

Time	0 min	1 min	2 min	4 min	6 min	8 min	10 min
nmol Glc-6P in assay	14.03	18.48	22.45	37.24	41.91	53.69	58.08
**Time**	**0 min**	**30 min**	**60 min**	**120 min**	**180 min**		
nmol Gal-6P in assay	N.D.	N.D.	N.D.	N.D.	N.D.		

An assay with Gal-1P was performed over an extended 3 h time period and samples taken at indicated time points. All values for Gal-6P are below the limit of detection of 35 fmol. Value of time point 0 min represents the formation of Glc-6P after addition of PGM3 and mixing the assay components before stopping the reaction with CHCl_3_/CH_3_OH (3:7, v/v). Control assays (without PGM3) did not show any formation of Glc-6P.

**Figure 6:**
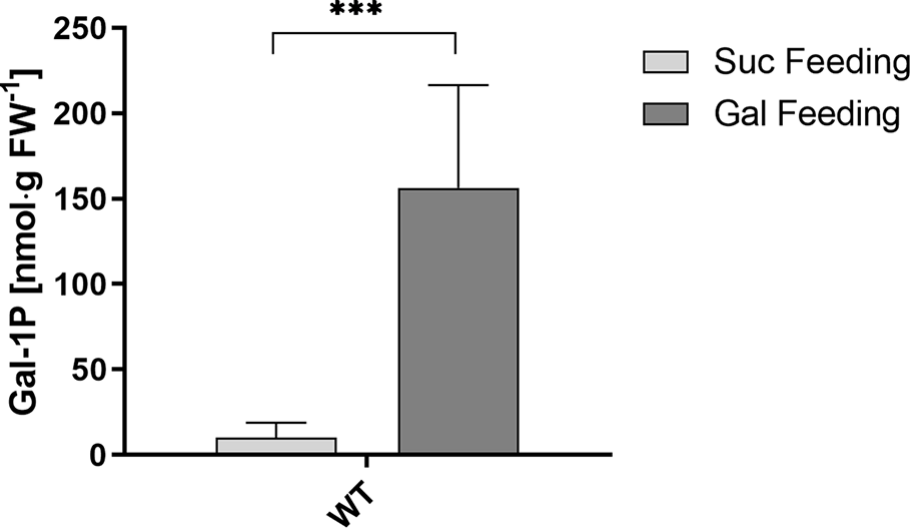
Gal-1P concentrations in root samples of WT seedlings. GC–MS measurements of Gal-1P levels in plants under Gal-feeding (5 mM Gal) versus Suc-feeding (5 mM Suc) conditions. Mean values (± SD) of three biological replicates with three technical replicates each are shown. Data analysis was performed using unpaired t-test ***: p < 0.001.

Data for sugar-phosphate levels in plants are frequently reported for Glc-1P and Glc-6P but we did not find published data for Gal-1P quantities. This prompted us to analyze the level of Gal-1P in WT plants, grown on MS-plates with either sucrose or galactose to determine physiological concentrations under Gal-feeding conditions. When extracting the ion current chromatogram for the quantifier ions at 205.99, 257.00, 298.93, 314.97, 387.04 and 428.95 *m/z* a small, yet distinguished peak for Gal-1P was detectable. To compensate for matrix effects on the derivatization due to the presence of other cellular metabolites, we performed absolute quantification using the standard addition method. Therefore, we split single metabolite samples into two equal portions and added Gal-1P at 35 pmol to one aliquot of the metabolite extracts. Due to the minute amounts available for root samples, only one spiked standard was used, however, independently repeated for three biological samples. Fortunately, derivatization of Gal-1P was not inhibited by metabolites from the plant sample, as metabolite samples spiked with Gal-1P gave almost identical peak areas as reference compounds of the same concentration. We subsequently used the integrated signals from GC-MS analyses to calculate the intracellular levels of Gal-1P. The concentration of Gal-1P in seedlings, grown on MS-plates with sucrose, was rather low (approx. 8 nmol·g^−1^ FW). When seedlings were grown on MS-plates in the presence of galactose, the amount of Gal-1P was strongly increased to approx. 160 nmol·g^−1^ FW (**Figure 6**). The physiological levels for Gal-1P that we found in cells were similar to the concentrations of Gal-1P which we used in the PGM assays, shown in **Figure 4**.

In metabolite extracts, Gal-6P was below the limit of detection at 35 fmol on column (**Table 1**). Given that Gal-1P was accumulated more than 20-fold in seedlings grown on Gal containing media, the analysis suggested that Gal-1P was not converted to Gal-6P *via* the enzyme PGM. This finding was in contrast to the data published on a yeast model by (De Jongh et al., 2008).

## Discussion

Gal-toxicity is widely found in nature but the mechanism of the toxicity is still unknown (Lai et al., 2009; Egert et al., 2012; Timson, 2016). One prominent hypothesis is that Gal-1P accumulates in cells upon Gal feeding and that the excess of Gal-1P inhibits the enzyme PGM. Here, we tested this hypothesis for Arabidopsis seedlings directly using PGM3 as a recombinant enzyme. Gal-1P levels in plants were rarely reported in the past so that data for plants are scarce. The amount of Gal-1P in plant metabolite extracts from seedlings grown on sucrose is rather low (approx. 8 nmol·g^−1^ FW) and comparable to levels in healthy human erythrocytes (Yuzyuk et al., 2018). We measured a strong increase in Arabidopsis under Gal-feeding and found Gal-1P levels of 150–200 to µmol·l^−1^, as observed for other organisms under conditions of mild Gal-toxicity (Yuzyuk et al., 2018). Yeast strains overexpressing galactose permease as well as galactose kinase accumulated Gal-1P at approx. 1 mmol·l^−1^ (Gibney et al., 2018). Similarly, Gal-1P levels between 1 and 5 mmol·l^−1^ were found in erythrocytes of humans, suffering from severe galactosemia (Gitzelmann, 1995). Sugarcane cell cultures were used in Gal feeding experiments by (Maretzki and Thom, 1978). In non-adapted cell lines an amount of 7.25 nmol·g^−1^ dry weight was found while Gal feeding, which roughly corresponds to 700 µmol·l^−1^. In this experiment, Gal was added at very high concentrations (100 mmol·l^−1^), whereas our Arabidopsis seedlings were exposed to only 1–10 mmol·l^−1^ Gal. A possible explanation for the lower Gal-1P levels under conditions of Gal-toxicity in plants compared to yeast and human might be a more efficient removal of Gal-1P into UDP-Gal by either galactose-1-uridyltransferase (Joersbo et al., 2003; Dai et al., 2006) or UDP-sugar pyrophosphorylase (Kotake et al., 2004; Geserick and Tenhaken, 2013). UDP-sugar pyrophosphorylase is a powerful enzyme and can build up high levels of UDP-arabinose in mutants with higher levels of arabinose-1P (Behmuller et al., 2016).

Toxicity of arabinose as well as of Gal share the necessity of a sugar-1-kinase. Knockout mutants in arabinokinase (Behmuller et al., 2016) or galactokinase (Egert et al., 2012) accumulate the free sugar to even high levels, but do not show the typical phenotype of toxicity.

Though arabinose and galactose are toxic for Arabidopsis, the phenotypes observed in the presence of the sugars are quite different. The phenotype of Gal toxicity mainly changes the root system. The leaves are unaffected at the morphological level. This can be explained when we assume that they already produce enough sucrose by photosynthesis, which typically represses the toxicity phenotype. In contrast, *ara1-1* mutants die on agar plates with 5 mM arabinose within 3 days (Behmuller et al., 2016). When young seedlings germinate on MS-medium and are later transferred to agar plates with arabinose, many of the seedlings will nevertheless survive. We interpret this observation that photosynthesis in cotyledons and primary leaves produce increasing amounts of sucrose, which blocks the toxicity phenotype. The mode of action by which sucrose prevents the toxicity phenotype is unfortunately unknown. Whether the sucrose effect acts *via* the same mechanism in Gal and arabinose toxicity needs to be addressed in future work.

An old suggested explanation concerns the possible depletion of cells from phosphate as the cause of sugar toxicity. The sugar-phosphate levels that we found under conditions of Gal-feeding were not very high and a possible phosphate depletion could easily be overcome by external phosphate addition. We and others tested this hypothesis by adding more phosphate (low mmol·l^−1^ concentration) to the growth medium. However, more phosphate did not revert the phenotype towards the wild type, thus disproving the phosphate deprivation hypothesis as the cause of Gal-toxicity.

### Gal-1P Is Not a Substrate for PGM3

One possibility, how Gal-1P might inhibit PGM, is the slow conversion of Gal-1P into Gal-6P. The catalytic cycle would involve the phosphorylation of the PGM enzyme forming Gal-1,6bP as a possible intermediate. In fact, (De Jongh et al., 2008) found slightly elevated levels of Gal-6P in yeast, overexpressing the uptake transporter *Gal2*. However, the data for yeast did not show a strong correlation between the level of Gal-1P and the specific growth rate and Gal-uptake rate. From this the authors concluded that Gal-1P cannot be the solely responsible factor for Gal-toxicity (De Jongh et al., 2008). A similar mode of PGM inhibition was shown for a β-PGM from *L. lactis* (Tremblay et al., 2005). Using recombinant Arabidopsis PGM3, we performed enzyme assays with Gal-1P as a substrate. Even after very long incubation times of up to 3 h, no detectable Gal-6P was found in the GC–MS analysis. Thus, it seems highly unlikely that the conversion of galactose-phosphates is a central determinant of galactose toxicity. A second mode of inhibition of PGM by Gal-1P might be the structural similarity to Glc-1P. In this case, we would expect a competitive inhibition of PGM by Gal-1P in an enzyme assay in the presence of Glc-1P. As shown in **Figure 4**, this inhibition does not occur. The experiments shown in this paper do not support the traditionally assumed hypothesis that inhibition of PGM is the major mode of action of Gal in sugar toxicity in plants. Nevertheless, knockout plants in galactokinase behave very similar to WT plants. This is a strong prove, that phosphorylation of Gal to Gal-1P is required to cause the observed sugar toxicity.

## Data Availability Statement

All datasets generated for this study are included in the article/**Supplementary Material**.

## Author Contributions

MA, CB, RR, CH, and RT designed the study and wrote the paper. CB, RR, and CH developed the analytical GC-MS procedures. RT cloned the expression construct. MA did most of the enzyme assays. MA and CB optimized the sugar phosphate analytics.

## Funding

This project was funded by a grant of the Austrian Science Fund (FWF; P25339) to RT.

## Conflict of Interest

The authors declare that the research was conducted in the absence of any commercial or financial relationships that could be construed as a potential conflict of interest.
