# Supplementary material for: Phosphoglucomutase Is Not the Target for Galactose Toxicity in Plants
Source: Front Plant Sci. 2020 Feb 28;11:167. doi: 10.3389/fpls.2020.00167 (PMC7059798; doi:10.3389/fpls.2020.00167)

Supplemental Data

Phosphoglucomutase is not the target for galactose toxicity in plants

Martina Althammer[1], Constantin Blöchl[2], Roland Reischl[2], Christian G. Huber[2], Raimund Tenhaken[1]

Supplemental Fig. 1

Lineweaver-Burk plot of PGM3 for the substrate Glc-1P (A) and the cosubstrate Glc-1,6bP. Enzyme Assays were performed at 25°C for 3 min. Values are averages of three independently performed assays (± SD)

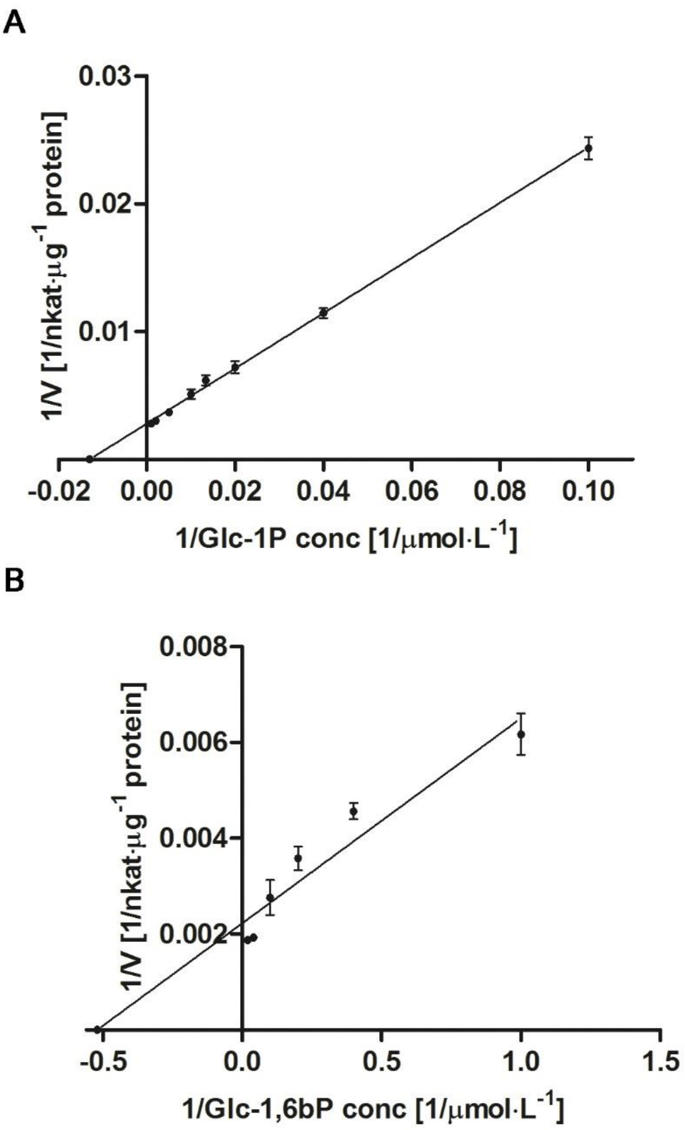

Supplement: Supplementary file 1 [file DataSheet_1.pdf]
